# Chloride of Calcium

**Published:** 1878-06

**Authors:** 


### ARTICLE VI.

### *Chloride of Calcium.*

Dr. Robert Bell thinks that this salt possesses much greater therapeutic value than has been accorded it, and though it has been crowded out by iodine and cod liver oil in the treatment of wasting diseases of childhood, he is satisfied it is often of incalculable service.

In tuburcular diseases of the bone sand joints of children, and in affections of the cervical glands, he has never met with its equal.

As it has been often suggested that lime is of therapeutic value in many cases where mal-nutrition (intra-uterine) is suspected,—cases where the anæmic condition of the pregrant woman would indicate the scarcity of lime salts, might not this salt, if as easily assimulated as Dr. Bell seems to think, be useful in such cases. We are more than ever convinced that the teeth of the coming and existing generations are of bad quality from defectrine intra uterine nourishment, and if it be true that the chloride of calcium is assimilable as suggested, it is worth while to try it in such cases; and the more so if the doctrine taught be true, that the teeth of the fœtus are built up at the expense of the mother.

